# Translation in fragile X: no home runs in the first at-bat

**DOI:** 10.1186/s11689-017-9204-y

**Published:** 2017-06-12

**Authors:** Jeremy Veenstra-VanderWeele

**Affiliations:** 1 Department of Psychiatry and Sackler Institute of Developmental Psychobiology, Columbia University, New York, NY USA; 2 New York State Psychiatric Institute, New York, NY USA; 3 Center for Autism and the Developing Brain, New York-Presbyterian Hospital, New York, NY USA; 4 1051 Riverside Drive, Mail Unit 78, New York, NY 10032 USA

Four articles in this issue of the *Journal* describe various steps in the cascade of research from gene discovery to potential treatment in fragile X syndrome (FXS) [1–4]. Objectively, FXS is one of the most productive areas of research in neurodevelopmental disorders. Twenty-six years ago, the cause of FXS was identified as a trinucleotide repeat expansion that disrupts *FMR1* gene expression [5]. This discovery quickly led to cellular and mouse models that lacked the corresponding protein, FMRP, leading in turn to fundamental insights into its function in the cell and at the synapse. Over the past 12 years, several groups have identified small molecules that rescue neuroanatomical, electrophysiological, and behavioral features in animal models of FXS. These results are tremendously exciting!

Subjectively, it has been harder to maintain an appropriate level of excitement. Based upon results in mouse models [6, 7], three pharmaceutical companies stepped up to test novel compounds in well-powered controlled trials in FXS. A couple of early pilot studies raised hopes further. Unfortunately, larger follow-up studies showed no significant findings in adults or adolescents with fragile X syndrome, including one study published in this issue of the *Journal* [2]. As a result, one of the three companies folded and the other two pulled out of FXS research.

We should not have expected that it would be simple to translate findings from a rodent model of FXS into humans. Clearly, the pharmaceutical industry has had tremendous success translating molecular findings in other areas, like breast cancer or HIV. Unfortunately, however, there are no examples of successful drug development programs in neurodevelopmental disorders that would indicate how best to test a potential drug for FXS. To draw on a sports analogy, this would be like a professional golfer picking up a baseball bat and expecting to hit a home run on his first at-bat. The initial programs for these three compounds were three swings to hit the ball over the fence. None of the programs hit a home run. The good news is that baseball games are not determined by the first at-bat.

The four articles in this issue offer us perspectives on the challenges and potential rewards of the translational game. One of these challenges is deciphering what model systems are telling us in relation to the human disorder. In this issue, Schaefer and colleagues [1] describe studies of acamprosate, which is approved by the Food and Drug Administration (FDA) for maintenance treatment of alcohol dependence. Acamprosate is not a simple drug. It binds to glutamatergic NMDA receptors and also seems to have mGluR5 and GABA-A effects. Likewise, the *Fmr1* null mouse is not a simple model. The authors use a different inbred mouse strain than in much of the literature and find no change in dendritic spine density in the *Fmr1* null compared to wildtype controls, despite this phenotype being a common target of rescue experiments on other inbred strain backgrounds. They do find an increase in synchronous neuronal firing in the somatosensory cortex, which is normalized by acamprosate. Likewise, they find that this drug rescues changes in ERK signaling and activity levels. These are important findings, but one of the key challenges in translating findings from mouse to human has been the lack of straightforward cognitive (or social) deficits in the *Fmr1* null mouse.

Another challenge to translational research in FXS has been that the first three large-scale clinical trial programs studied adolescents and adults, rather than aiming to treat this neurodevelopmental disorder during childhood. In this issue, Berry-Kravis and colleagues describe two randomized, placebo-controlled trials of arbaclofen [2], a GABA-B agonist, that follow up suggestive findings in a phase 2 randomized, cross-over study [8]. The results of the larger study, a two-group trial of one dose versus placebo in adults and adolescents (*n* = 125), parallel the negative findings in trials of mGluR5 negative allosteric modulators in adults and adolescents [9]. Unfortunately, the four-group study of three different doses versus placebo in children (*n* = 172) was cut short when the study sponsor went out of business. However, this trial shows some tantalizing hints of response, including improvements in measures of irritability and parenting stress. We should not over-interpret these preliminary results in a limited sample, but they do suggest that we may see more impact of treatment in children with FXS than in adolescents and adults. Unfortunately, the FDA approval process typically follows a cascade from adults to adolescents to children.

Budimirovic and colleagues tackle another challenge in FXS translational research: the absence of well-validated, reliable outcome measures that are sensitive to treatment in FXS [3]. As they note, it is difficult to establish a gold standard outcome measure without a gold standard treatment that has shown efficacy in controlled trials. To identify the most promising outcome measures to date, they conducted a systematic analysis of outcome measures used in controlled trials. Based upon the existing literature, they graded several instruments at a moderate tool quality rating, suggesting that they may be useful for inclusion in future studies. Two measures were scored at a moderate-to-strong level, likely the best that can be achieved without definitive clinical trials that would lead to an FDA indication. Neither of these measures is a comprehensive assessment of the fragile X phenotype. The Test of Attentional Performance in Children (KiTAP) is a computerized measure of executive function that may still exclude some lower-functioning children. The Aberrant Behavior Checklist, Community version, modified for fragile X syndrome, (ABC-C_FX_), is a parent report measure that emphasizes maladaptive behavior. Budimirovic and colleagues conclude by highlighting the need for continued development of objective measures of cognitive function, as well as potential biomarkers of treatment response.

Finally, Erickson and colleagues review the lessons learned from previous treatment studies in FXS, with an eye toward the design of future studies [4]. They highlight a number of the key issues outlined above, including disconnects between mouse and human phenotypes, an FDA-imposed focus on adults and adolescents rather than children, and outcome measures that are subjective or adapted from the other neurodevelopmental conditions, such as autism spectrum disorder. Recognizing what has not worked in FXS trials, they call for treatment studies in children, trial durations that last long enough to assess improvements in adaptive function, and measurement of outcomes that are core deficits in FXS, such as cognition and communication. They also urge us to be careful in drawing conclusions from findings that fall short of statistical significance after correcting for multiple analyses, believing that such findings should be only judiciously used to design future studies.

If you have not watched a lot of baseball games, it is easy to be disappointed when the first batter strikes out, or even when there are no runs scored in the first inning. Taking a nine-inning view, however, there is considerable reason for optimism in FXS. Erickson and colleagues note the tremendous increase in knowledge over the past decade, both with regard to the neurobiological impact of FMRP loss, at least in mice, and with regard to how to conduct FXS treatment studies. While the first few swings at FXS treatment did not generate home runs, they did develop a clinical trial infrastructure that has drawn other industry players to the FXS field. Even beyond finding neurobiologically informed treatments for FXS, we are learning how the translational game works, with the hope that the knowledge gained will also teach us how to test treatments in other neurodevelopmental disorders.
